# Supplementary material for: Evaluation of fetal exposure to environmental noise using a computer-generated model
Source: Nat Commun. 2025 Apr 25;16:3916. doi: 10.1038/s41467-025-58983-0 (PMC12032418; doi:10.1038/s41467-025-58983-0)
Supplement: Supplementary file 1 — Supplementary Information [file 41467_2025_58983_MOESM1_ESM.pdf]

Supplementary Information - Mathematical formulation

1 The computer-generated model

The main text evaluates the fetal exposure to external noise using a computer-generated model. This supplementary information provides a detailed presentation of the mathematical formulations underpinning the computer-generated model.

2 Model equations

Let us consider acoustic wave propagation through the entire three-dimensional space, composed of four homogeneous domains, each denoted by Ω_m, for $m \in \{0, 1, 2, 3\}$. The domain Ω_0 is the unbounded exterior medium, Ω_1 a bounded domain with Ω_2 and Ω_3 inside. See Figure 1 for a sketch of the geometry. There are three material interfaces: Γ_1 between Ω_0 and Ω_1, Γ_2 between Ω_1 and Ω_2, and Γ_3 between Ω_1 and Ω_3. All surfaces are smooth and the unit normals $\hat{\mathbf{n}}_m$ corresponding to surface Γ_m all point towards the exterior.

We consider harmonic wave fields with an $e^{-\imath\omega t}$ time dependency and a fixed frequency f, where $\omega = 2\pi f$ denotes the angular frequency. Each material domain Ω_m has a constant density and speed of sound, denoted by ρ_m and c_m, respectively. The wavenumber is $k_m = 2\pi f/c_m + \imath\alpha_m$ where α_m denotes the attenuation coefficient that may depend on the frequency. The

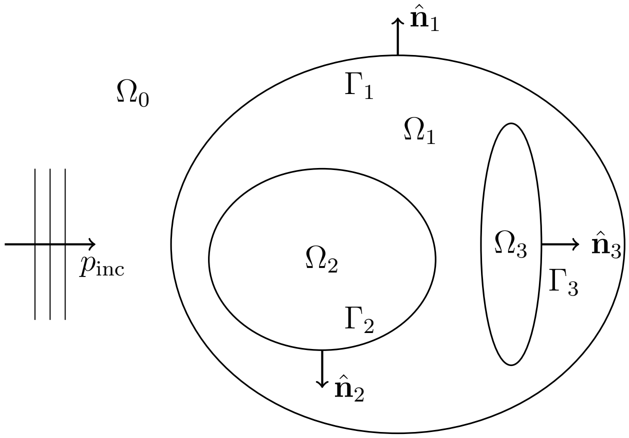

Figure 1: A sketch of the geometry of the model.

Helmholtz system

$$\begin{cases} -\nabla^2 p_m - k_m^2 p_m = 0, & \text{in } \Omega_m \text{ for } m \in \{0,1,2,3\}; \\ p_0 + p_{\text{inc}} = p_1, & \text{at } \Gamma_1; \\ p_1 = p_n, & \text{at } \Gamma_n \text{ for } n \in \{2,3\}; \\ \frac{1}{\rho_0}\frac{\partial}{\partial \hat{\mathbf{n}}_1}(p_0 + p_{\text{inc}}) = \frac{1}{\rho_1}\frac{\partial}{\partial \hat{\mathbf{n}}_1} p_1, & \text{at } \Gamma_1; \\ \frac{1}{\rho_1}\frac{\partial}{\partial \hat{\mathbf{n}}_n} p_1 = \frac{1}{\rho_n}\frac{\partial}{\partial \hat{\mathbf{n}}_n} p_n, & \text{at } \Gamma_n \text{ for } n \in \{2,3\}; \\ \lim_{\mathbf{r}\to\infty} |\mathbf{r}| \left(\partial_{|\mathbf{r}|} p_0 - \imath k_0 p_0\right) = 0; \end{cases} \tag{1}$$

models the harmonic wave propagation. The second until fifth equation are the interface conditions stating continuity of the pressure field and the particle velocity in normal direction. The sixth equation is the Sommerfeld radiation condition for outgoing waves. Here,

$$p_0(\mathbf{x}) = \begin{cases} p_{\text{sca}}(\mathbf{x}), & \mathbf{x} \in \Omega_0; \\ -p_{\text{inc}}(\mathbf{x}), & \mathbf{x} \in \Omega_m \text{ for } m \in \{1,2,3\}; \end{cases} \tag{2}$$

represents the unknown scattered field while

$$p_m(\mathbf{x}) = \begin{cases} p_{\text{tot}}(\mathbf{x}), & \mathbf{x} \in \Omega_m; \\ 0, & \mathbf{x} \in \Omega_n \text{ for } n \in \{0,1,2,3\} \setminus \{m\}; \end{cases} \tag{3}$$

represent the unknown pressure field in the interior domains Ω_m for $m \in$

$\{1, 2, 3\}$. The incident field $p_{\text{inc}} = p_{\text{tot}} - p_{\text{sca}}$ is a plane wave field

$$p_{\text{inc}}(\mathbf{x}) = A e^{\imath k_0 \mathbf{x} \cdot \hat{\mathbf{d}}}, \quad \mathbf{x} \in \mathbb{R}^3; \tag{4}$$

of amplitude A and travelling in the direction $\hat{\mathbf{d}}$.

3 Boundary integral formulations

The Green's functions

$$G_m(\mathbf{x}, \mathbf{y}) = \frac{e^{\imath k_m |\mathbf{x}-\mathbf{y}|}}{4\pi |\mathbf{x}-\mathbf{y}|} \quad \text{for } \mathbf{x} \neq \mathbf{y} \tag{5}$$

are the fundamental solutions of the Helmholtz equations in each homogeneous subdomain Ω_m for $m \in \{0, 1, 2, 3\}$. They provide the building blocks for the reformulation of the Helmholtz equation in each subdomain Ω_m to boundary integral equations at the surfaces Γ_n. This procedure is well documented for impenetrable and single penetrable domains, cf. [3, 7, 5]. However, the formulation requires a new design for our targeted geometry, shown in Figure 1.

Let us consider the direct representation formulas

$$p_0(\mathbf{x}) = \mathcal{K}_{0,1}\phi_{0,1} - \mathcal{V}_{0,1}\psi_{0,1}, \qquad \mathbf{x} \notin \Gamma_1; \tag{6}$$

$$\begin{aligned} p_1(\mathbf{x}) = {} & \mathcal{V}_{1,1}\psi_{1,1} - \mathcal{K}_{1,1}\phi_{1,1} \\ & + \mathcal{K}_{1,2}\phi_{1,2} - \mathcal{V}_{1,2}\psi_{1,2} + \mathcal{K}_{1,3}\phi_{1,3} - \mathcal{V}_{1,3}\psi_{1,3}, \qquad \mathbf{x} \notin \{\Gamma_1, \Gamma_2, \Gamma_3\}; \end{aligned} \tag{7}$$

$$p_2(\mathbf{x}) = \mathcal{V}_{2,2}\psi_{2,2} - \mathcal{K}_{2,2}\phi_{2,2}, \qquad \mathbf{x} \notin \Gamma_2; \tag{8}$$

$$p_3(\mathbf{x}) = \mathcal{V}_{3,3}\psi_{3,3} - \mathcal{K}_{3,3}\phi_{3,3}, \qquad \mathbf{x} \notin \Gamma_3; \tag{9}$$

that relate the volumetric pressure fields p_m for $m \in \{0, 1, 2, 3\}$ with the Dirichlet and Neumann surface potentials $\phi_{m,n}$ and $\psi_{m,n}$ at Γ_n for $n \in \{1, 2, 3\}$. Here,

$$[\mathcal{V}_{m,n}\psi](\mathbf{x}) = \iint_{\Gamma_n} G_m(\mathbf{x}, \mathbf{y})\psi(\mathbf{y})\, \mathrm{d}\mathbf{y} \qquad \text{for } \mathbf{x} \notin \Gamma_n; \tag{10}$$

$$[\mathcal{K}_{m,n}\phi](\mathbf{x}) = \iint_{\Gamma_n} \frac{\partial}{\partial \hat{\mathbf{n}}_n(\mathbf{y})} G_m(\mathbf{x}, \mathbf{y})\phi(\mathbf{y})\, \mathrm{d}\mathbf{y} \qquad \text{for } \mathbf{x} \notin \Gamma_n; \tag{11}$$

the single-layer and double-layer potential operators, respectively.

To achieve model equations formulated entirely on the material interfaces, the limits of the representation formula towards the surfaces need to be taken. Hence, let us define

$$\gamma^D_{m,n} f(\mathbf{x}) = \lim_{\mathbf{y}\to\mathbf{x}} f(\mathbf{y}) \qquad \text{for } \mathbf{x} \in \Gamma_n \text{ and } \mathbf{y} \in \Omega_m, \tag{12}$$

$$\gamma^N_{m,n} f(\mathbf{x}) = \lim_{\mathbf{y}\to\mathbf{x}} \nabla f(\mathbf{y}) \cdot \hat{\mathbf{n}}_n(\mathbf{x}) \qquad \text{for } \mathbf{x} \in \Gamma_n \text{ and } \mathbf{y} \in \Omega_m, \tag{13}$$

the Dirichlet and Neumann traces, respectively, for Ω_m a subdomain that neighbors surface Γ_n.

Furthermore, let us define

$$[V_{m,pq}\psi](\mathbf{x}) = \iint_{\Gamma_q} G_m(\mathbf{x},\mathbf{y})\psi(\mathbf{y})\,\mathrm{d}\mathbf{y} \qquad \text{for } \mathbf{x} \in \Gamma_p, \tag{14}$$

$$[K_{m,pq}\phi](\mathbf{x}) = \iint_{\Gamma_q} \frac{\partial}{\partial \hat{\mathbf{n}}_q(\mathbf{y})} G_m(\mathbf{x},\mathbf{y})\phi(\mathbf{y})\,\mathrm{d}\mathbf{y} \qquad \text{for } \mathbf{x} \in \Gamma_p, \tag{15}$$

$$[T_{m,pq}\psi](\mathbf{x}) = \frac{\partial}{\partial \hat{\mathbf{n}}_p(\mathbf{x})} \iint_{\Gamma_q} G_m(\mathbf{x},\mathbf{y})\psi(\mathbf{y})\,\mathrm{d}\mathbf{y} \qquad \text{for } \mathbf{x} \in \Gamma_p, \tag{16}$$

$$[D_{m,pq}\phi](\mathbf{x}) = -\frac{\partial}{\partial \hat{\mathbf{n}}_p(\mathbf{x})} \iint_{\Gamma_q} \frac{\partial}{\partial \hat{\mathbf{n}}_q(\mathbf{y})} G_m(\mathbf{x},\mathbf{y})\phi(\mathbf{y})\,\mathrm{d}\mathbf{y} \qquad \text{for } \mathbf{x} \in \Gamma_p, \tag{17}$$

the single-layer, double-layer, adjoint double-layer, and hypersingular boundary integral operators, respectively. Since direct representation formulas were chosen, the surface potentials are given by the traces of the pressure field [8]. Precisely,

$$\phi_{m,n} = \gamma^D_{m,n} p_{\mathrm{tot}}, \tag{18}$$

$$\psi_{m,n} = \gamma^N_{m,n} p_{\mathrm{tot}}. \tag{19}$$

Hence, we can define unique pairs of surface potentials at each surface as

$$\phi_1 = \phi_{0,1} = \gamma^D_{0,1} p_{\mathrm{tot}} = \gamma^D_{1,1} p_{\mathrm{tot}} = \phi_{1,1}, \tag{20}$$

$$\psi_1 = \psi_{0,1} = \gamma^N_{0,1} p_{\mathrm{tot}} = \frac{\rho_0}{\rho_1}\gamma^N_{1,1} p_{\mathrm{tot}} = \frac{\rho_0}{\rho_1}\psi_{1,1}, \tag{21}$$

$$\phi_2 = \phi_{1,2} = \gamma^D_{1,2} p_{\mathrm{tot}} = \gamma^D_{2,2} p_{\mathrm{tot}} = \phi_{2,2}, \tag{22}$$

$$\psi_2 = \psi_{1,2} = \gamma^N_{1,2} p_{\mathrm{tot}} = \frac{\rho_1}{\rho_2}\gamma^N_{1,2} p_{\mathrm{tot}} = \frac{\rho_1}{\rho_2}\psi_{2,2}, \tag{23}$$

$$\phi_3 = \phi_{1,3} = \gamma^D_{1,3} p_{\mathrm{tot}} = \gamma^D_{2,3} p_{\mathrm{tot}} = \phi_{2,3}, \tag{24}$$

$$\psi_3 = \psi_{1,3} = \gamma^N_{1,3} p_{\mathrm{tot}} = \frac{\rho_1}{\rho_3}\gamma^N_{1,3} p_{\mathrm{tot}} = \frac{\rho_1}{\rho_3}\psi_{3,3}, \tag{25}$$

because of the interface conditions.

Taking the traces of the representation formulas yields equations for surface operators and potentials only. Specifically,

$$\begin{bmatrix} -K_{1,11} - K_{0,11} & \frac{\rho_1}{\rho_0}V_{1,11} + V_{0,11} & K_{1,12} & -V_{1,12} & K_{1,13} & -V_{1,13} \\ \frac{\rho_0}{\rho_1}D_{1,11} + D_{0,11} & T_{1,11} + T_{0,11} & -\frac{\rho_0}{\rho_1}D_{1,12} & -\frac{\rho_0}{\rho_1}T_{1,12} & -\frac{\rho_0}{\rho_1}D_{1,13} & -\frac{\rho_0}{\rho_1}T_{1,13} \\ K_{1,21} & -\frac{\rho_1}{\rho_0}V_{1,21} & -K_{2,22} - K_{1,22} & \frac{\rho_2}{\rho_1}V_{2,22} + V_{1,22} & -K_{1,23} & V_{1,23} \\ -D_{1,21} & -\frac{\rho_1}{\rho_0}T_{1,21} & \frac{\rho_1}{\rho_2}D_{2,22} + D_{1,22} & T_{2,22} + T_{1,22} & D_{1,23} & T_{1,23} \\ K_{1,31} & -\frac{\rho_1}{\rho_0}V_{1,31} & -K_{1,32} & V_{1,32} & -K_{3,33} - K_{1,33} & \frac{\rho_3}{\rho_1}V_{3,33} + V_{1,33} \\ -D_{1,31} & -\frac{\rho_1}{\rho_0}T_{1,31} & D_{1,32} & T_{1,32} & \frac{\rho_1}{\rho_3}D_{3,33} + D_{1,33} & T_{3,33} + T_{1,33} \end{bmatrix} \begin{bmatrix} \phi_1 \\ \psi_1 \\ \phi_2 \\ \psi_2 \\ \phi_3 \\ \psi_3 \end{bmatrix} = \begin{bmatrix} \gamma_{0,1}^D p_{\text{inc}} \\ \gamma_{0,1}^N p_{\text{inc}} \\ 0 \\ 0 \\ 0 \\ 0 \end{bmatrix} \tag{26}$$

the boundary integral equation. This equation is a single-trace direct boundary integral formulation similar to the Poggio-Miller-Chang-Harrington-Wu-Tsai (PMCHWT) and Costabel-Stephan formulations [4, 1, 10, 2] for a single penetrable domain but adapted to the geometry in Figure 1.

4 Boundary element method

The boundary integral operators in the continous formulation (26) are discretized by a standard Galerkin method with local piecewise linear (P1) basis and test functions on a triangular surface mesh [6]. The conditioning of the discrete system is improved by pre-multiplying the equation with

$$\begin{bmatrix} 0 & \Lambda_1^{\text{NtD}} & 0 & 0 & 0 & 0 \\ \Lambda_1^{\text{DtN}} & 0 & 0 & 0 & 0 & 0 \\ 0 & 0 & 0 & \Lambda_2^{\text{NtD}} & 0 & 0 \\ 0 & 0 & \Lambda_2^{\text{DtN}} & 0 & 0 & 0 \\ 0 & 0 & 0 & 0 & 0 & \Lambda_3^{\text{NtD}} \\ 0 & 0 & 0 & 0 & \Lambda_3^{\text{DtN}} & 0 \end{bmatrix} \tag{27}$$

where Λ_n^{NtD} and Λ_n^{DtN} are Neumann-to-Dirichlet and Dirichlet-to-Neumann operators on surface Γ_n, respectively, and approximated with On-Surface

Radiation Conditions (OSRC). See [9] for the definitions of these operators and the numerical procedure to evaluate them. The final system will be solved by the Generalized Minimal Residual (GMRES) algorithm.

References

[1] Yu Chang and Roger F Harrington. A surface formulation for characteristic modes of material bodies. Technical report, Syracuse University, Syracuse, NY, 10 1974. Technical Report TR-74-7.

[2] Martin Costabel and Ernst Stephan. A direct boundary integral equation method for transmission problems. *Journal of Mathematical Analysis and Applications*, 106(2):367–413, 1985.

[3] Jean-Claude Nédélec. *Acoustic and electromagnetic equations: integral representations for harmonic problems*, volume 144 of *Applied Mathematical Sciences*. Springer, New York, 2001.

[4] A. J. Poggio and E. K. Miller. Integral equation solutions of three-dimensional scattering problems. In R. Mittra, editor, *Computer Techniques for Electromagnetics*, International Series of Monographs in Electrical Engineering, chapter 4, pages 159–264. Pergamon, Oxford, UK, 1973.

[5] Stefan A Sauter and Christoph Schwab. *Boundary Element Methods*, volume 39 of *Springer Series in Computational Mathematics*. Springer, Berlin, 2011.

[6] Wojciech Śmigaj, Timo Betcke, Simon Arridge, Joel Phillips, and Martin Schweiger. Solving boundary integral problems with BEM++. *ACM Transactions on Mathematical Software (TOMS)*, 41(2):6, 2015.

[7] Olaf Steinbach. *Numerical approximation methods for elliptic boundary value problems: finite and boundary elements*. Springer, New York, 2008.

[8] Elwin van 't Wout, Seyyed R. Haqshenas, Pierre Gélat, Timo Betcke, and Nader Saffari. Benchmarking preconditioned boundary integral formulations for acoustics. *International Journal for Numerical Methods in Engineering*, 122(20):5873–5897, 2021.

[9] Elwin van 't Wout, Seyyed R. Haqshenas, Pierre Gélat, Timo Betcke, and Nader Saffari. Frequency-robust preconditioning of boundary integral equations for acoustic transmission. *Journal of Computational Physics*, 462:111229, 2022.

[10] Te-Kao Wu and Leonard L Tsai. Scattering from arbitrarily-shaped lossy dielectric bodies of revolution. *Radio Science*, 12(5):709–718, 1977.
