# Supplementary material for: Evaluation of fetal exposure to environmental noise using a computer-generated model
Source: Nat Commun. 2025 Apr 25;16:3916. doi: 10.1038/s41467-025-58983-0 (PMC12032418; doi:10.1038/s41467-025-58983-0)
Supplement: Supplementary file 2 — Description of Additional Supplementary Files [file 41467_2025_58983_MOESM2_ESM.pdf]

Description of Additional Supplementary Files

Supplementary Audio 1: Reference soundscape generated from a range of audio signals which feature, in chronological sequence: (a) a London Underground train leaving and arriving at a station[54]; (b)
a segment of an instrumental ambient rock music composition[55]; (c) Ambient crow noise obtained from the Louvre museum[54] and (d) crowd applause[54].

Supplementary Audio 2: Reference soundscape (Supplementary Audio 1) filtered by a causal, linear and time-invariant filter was from in utero calculations on the dataset associated with Subjects 2, for the pressure at the barycentre of the uterus using the attenuation coefficient of uterine tissue (high attenuation case). The reference soundscape was convolved with this filter to yield an impression of in utero sound transmission.

Supplementary Audio 3: Reference soundscape (Supplementary Audio 1) filtered by a causal, linear and time-invariant filter was from in utero calculations on the dataset associated with Subjects 3, for the pressure at the barycentre of the uterus using the attenuation coefficient of uterine tissue (high attenuation case). The reference soundscape was convolved with this filter to yield an impression of in utero sound transmission.
