# Supplementary material for: Evaluation of fetal exposure to environmental noise using a computer-generated model
Source: Nat Commun. 2025 Apr 25;16:3916. doi: 10.1038/s41467-025-58983-0 (PMC12032418; doi:10.1038/s41467-025-58983-0)
Supplement: Supplementary file 6 — Reporting Summary [file 41467_2025_58983_MOESM6_ESM.pdf]

nature portfolio

Corresponding author(s): Dr Pierre Gélat

Last updated by author(s): 2025/02/14

Reporting Summary

Nature Portfolio wishes to improve the reproducibility of the work that we publish. This form provides structure for consistency and transparency in reporting. For further information on Nature Portfolio policies, see our Editorial Policies and the Editorial Policy Checklist.

Statistics

For all statistical analyses, confirm that the following items are present in the figure legend, table legend, main text, or Methods section.

n/a	Confirmed	
☒	☐	The exact sample size (*n*) for each experimental group/condition, given as a discrete number and unit of measurement
☒	☐	A statement on whether measurements were taken from distinct samples or whether the same sample was measured repeatedly
☒	☐	The statistical test(s) used AND whether they are one- or two-sided<br>*Only common tests should be described solely by name; describe more complex techniques in the Methods section.*
☒	☐	A description of all covariates tested
☒	☐	A description of any assumptions or corrections, such as tests of normality and adjustment for multiple comparisons
☒	☐	A full description of the statistical parameters including central tendency (e.g. means) or other basic estimates (e.g. regression coefficient) AND variation (e.g. standard deviation) or associated estimates of uncertainty (e.g. confidence intervals)
☒	☐	For null hypothesis testing, the test statistic (e.g. *F*, *t*, *r*) with confidence intervals, effect sizes, degrees of freedom and *P* value noted<br>*Give P values as exact values whenever suitable.*
☒	☐	For Bayesian analysis, information on the choice of priors and Markov chain Monte Carlo settings
☒	☐	For hierarchical and complex designs, identification of the appropriate level for tests and full reporting of outcomes
☒	☐	Estimates of effect sizes (e.g. Cohen's *d*, Pearson's *r*), indicating how they were calculated

Our web collection on statistics for biologists contains articles on many of the points above.

Software and code

Policy information about availability of computer code

Data collection	OptimUS v0.2.1; MATLAB 2024b; Meshmixer3.5; Gmsh 4.13.1
Data analysis	OptimUS v0.2.1; MATLAB 2024b; Meshmixer3.5; Gmsh 4.13.1

For manuscripts utilizing custom algorithms or software that are central to the research but not yet described in published literature, software must be made available to editors and reviewers. We strongly encourage code deposition in a community repository (e.g. GitHub). See the Nature Portfolio guidelines for submitting code & software for further information.

Data

Policy information about availability of data

All manuscripts must include a data availability statement. This statement should provide the following information, where applicable:

- Accession codes, unique identifiers, or web links for publicly available datasets
- A description of any restrictions on data availability
- For clinical datasets or third party data, please ensure that the statement adheres to our policy

The segmentation files derived from the MRI datasets employed in this study are available from the corresponding author upon reasonable request.
The code in this paper along with sample Jupyter notebooks is available from the GitHub repository https://github.com/optimuslib. The sample notebook demonstrating single frequency simulations in this paper can be found in the repository. Any data in this paper not published on this repository or available with this paper is available from the corresponding author upon reasonable request.

Research involving human participants, their data, or biological material

Policy information about studies with human participants or human data. See also policy information about sex, gender (identity/presentation), and sexual orientation and race, ethnicity and racism.

Reporting on sex and gender	The four datasets employed in this study were derived from MRI scans of individuals of pregnant individuals of female biological sex. We were not powered to investigate differences by fetal sex and we do not anticipate any fetal-sex-dependent differences in the study that we conducted.
Reporting on race, ethnicity, or other socially relevant groupings	Data was obtained from four individuals as part of a proof-of-concept study. We do not have sufficient power to investigate the effect of race, ethnicity or other social groupings.
Population characteristics	MRI was obtained from four pregnant individuals with appropriately developing pregnancies between 24 and 31 weeks of gestation.
Recruitment	Participants were recruited as part of the GIFT-SURG study: All MRI data were analysed under the study entitled ''Guided Instrumentation for Fetal Therapy and Surgery (GIFT-Surg): Fetal MRI to Improve Prenatal Diagnosis and Therapy for Fetal Abnormality'' (Hampstead Research Ethics Committee, 15/LO/1488), funded by the Wellcome Trust (210182/Z/18/Z) and EPSRC (NS/A000027/1). Women provided written informed consent for fetal MRI research. All images were transferred with Caldicott Guardian approval from University College London Hospitals (UCLH) to collaborators at partner academic institutions (University College London (UCL), King's College (KCL)) via the secure GIFT-Cloud platform, which ensures de-identification through XNAT technology. Images were chosen based on having good image quality and from a range of gestational ages. Only control pregnancies were considered.
Ethics oversight	The study was approved by the UK National Research Ethics Service and all participants gave written informed consent (London – Hampstead Research Ethics Committee, REC reference 15/LO/1488).

Note that full information on the approval of the study protocol must also be provided in the manuscript.

Field-specific reporting

Please select the one below that is the best fit for your research. If you are not sure, read the appropriate sections before making your selection.

☐ Life sciences ☐ Behavioural & social sciences ☒ Ecological, evolutionary & environmental sciences

For a reference copy of the document with all sections, see nature.com/documents/nr-reporting-summary-flat.pdf

Ecological, evolutionary & environmental sciences study design

All studies must disclose on these points even when the disclosure is negative.

Study description	Using anatomical data from MRI scans (N=4), with pregnancies for gestational age greater than 24 weeks, we used a computational model to quantify the acoustic field inside the pregnant maternal abdomen. We obtained acoustic transfer characteristics across the human audio range and pressure maps in transverse planes passing through the uterus at 5 kHz, 10 kHz and 20 kHz, showcasing multiple scattering and modal patterns.
Research sample	MRI scans of four patients with pregnancies above 24 weeks gestational age.
Sampling strategy	We were interested in subjects of 25-38 weeks gestational age with uncomplicated pregnancy.
Data collection	MRI examinations were performed on a 1.5 T magnet (MAGENETOM Avanto; Siemens Healthcare). Four women beyond 24 weeks of gestational age (confirmed by dating scan) with uncomplicated pregnancies had MRI data acquired. Patients were placed in the left lateral decubitus position and had a moderately filled bladder. The uterus was imaged in at least 3 orthogonal planes (axial, coronal, and sagittal) relative to the placenta-myometrium interface.
Timing and spatial scale	The protocol consisted of T2-weighted fast acquisition spin echo sequences (HASTE). The following parameters were applied: slice thickness (4 mm), spacing between slices (4 mm), repetition time (1013.8 ms), echo time (89.6 ms), flip angle (107.9°), and pixel spacing (0.74–0.74 mm).
Data exclusions	Images were chosen based on image quality and across a range of relevant gestational ages. Only control pregnancies were considered. We did not exclude any subjects after this choice was made.
Reproducibility	The study was entirely in silico. We have provided a means of model validation based on analytical solutions.
Randomization	This study was concerned with predicting in utero sound propagation on 4 samples. Randomisation was not required.
Blinding	Data were fully anonymised prior to analysis and no further blinding was necessary due to the nature of the study.

Did the study involve field work? ☐ Yes ☒ No

Reporting for specific materials, systems and methods

We require information from authors about some types of materials, experimental systems and methods used in many studies. Here, indicate whether each material, system or method listed is relevant to your study. If you are not sure if a list item applies to your research, read the appropriate section before selecting a response.

Materials & experimental systems

n/a	Involved in the study
☒	☐ Antibodies
☒	☐ Eukaryotic cell lines
☒	☐ Palaeontology and archaeology
☒	☐ Animals and other organisms
☐	☒ Clinical data
☒	☐ Dual use research of concern
☒	☐ Plants

Methods

n/a	Involved in the study
☒	☐ ChIP-seq
☒	☐ Flow cytometry
☒	☐ MRI-based neuroimaging

Clinical data

Policy information about clinical studies

All manuscripts should comply with the ICMJEguidelines for publication of clinical research and a completedCONSORT checklist must be included with all submissions.

Clinical trial registration	*Provide the trial registration number from ClinicalTrials.gov or an equivalent agency.*
Study protocol	*Note where the full trial protocol can be accessed OR if not available, explain why.*
Data collection	*Describe the settings and locales of data collection, noting the time periods of recruitment and data collection.*
Outcomes	*Describe how you pre-defined primary and secondary outcome measures and how you assessed these measures.*

Plants

Seed stocks	*Report on the source of all seed stocks or other plant material used. If applicable, state the seed stock centre and catalogue number. If plant specimens were collected from the field, describe the collection location, date and sampling procedures.*
Novel plant genotypes	*Describe the methods by which all novel plant genotypes were produced. This includes those generated by transgenic approaches, gene editing, chemical/radiation-based mutagenesis and hybridization. For transgenic lines, describe the transformation method, the number of independent lines analyzed and the generation upon which experiments were performed. For gene-edited lines, describe the editor used, the endogenous sequence targeted for editing, the targeting guide RNA sequence (if applicable) and how the editor was applied.*
Authentication	*Describe any authentication procedures for each seed stock used or novel genotype generated. Describe any experiments used to assess the effect of a mutation and, where applicable, how potential secondary effects (e.g. second site T-DNA insertions, mosiacism, off-target gene editing) were examined.*
